# Metal-organic frameworks bonded with metal *N*-heterocyclic carbenes for efficient catalysis

**DOI:** 10.1093/nsr/nwab157

**Published:** 2021-08-24

**Authors:** Chang He, Jun Liang, Yu-Huang Zou, Jun-Dong Yi, Yuan-Biao Huang, Rong Cao

**Affiliations:** State Key Laboratory of Structural Chemistry, Fujian Institute of Research on the Structure of Matter, Chinese Academy of Sciences, Fuzhou 350002, China; University of Chinese Academy of Sciences, Beijing 100049, China; State Key Laboratory of Structural Chemistry, Fujian Institute of Research on the Structure of Matter, Chinese Academy of Sciences, Fuzhou 350002, China; State Key Laboratory of Structural Chemistry, Fujian Institute of Research on the Structure of Matter, Chinese Academy of Sciences, Fuzhou 350002, China; State Key Laboratory of Structural Chemistry, Fujian Institute of Research on the Structure of Matter, Chinese Academy of Sciences, Fuzhou 350002, China; State Key Laboratory of Structural Chemistry, Fujian Institute of Research on the Structure of Matter, Chinese Academy of Sciences, Fuzhou 350002, China; University of Chinese Academy of Sciences, Beijing 100049, China; State Key Laboratory of Structural Chemistry, Fujian Institute of Research on the Structure of Matter, Chinese Academy of Sciences, Fuzhou 350002, China; University of Chinese Academy of Sciences, Beijing 100049, China; Science and Technology Innovation Laboratory for Optoelectronic Information of China, Fuzhou 350108, China

**Keywords:** metal-organic framework, *N*-heterocyclic carbene, MIL-101, Suzuki reaction, transfer hydrogenation

## Abstract

Metal *N-*heterocyclic carbenes (M-NHCs) on the pore walls of a porous metal-organic framework (MOF) can be used as active sites for efficient organic catalysis. Traditional approaches that need strong alkaline reagents or insoluble Ag_2_O are not, however, suitable for the incorporation of NHCs on the backbones of MOFs because such reagents could destroy their frameworks or result in low reactivity. Accordingly, development of facile strategies toward functional MOFs with covalently bound M-NHCs for catalysis is needed. Herein, we describe the development of a general and facile approach to preparing MOFs with covalently linked active M-NHC (M = Pd, Ir) single-site catalysts by using a soluble Ag salt AgOC(CF_3_)_3_ as the source and subsequent transmetalation. The well-defined M-NHC-MOF (M = Pd, Ir) catalysts obtained in this way have shown excellent catalytic activity and stability in Suzuki reactions and hydrogen transfer reactions. This provides a general and facile strategy for anchoring functional M-NHC single-site catalysts onto functionalized MOFs for different reactions.

## Teaser

A unique strategy is developed to fabricate metal-carbene-decorated MOFs for efficient catalysis by using a soluble silver source and a subsequent transmetalation reaction, which would be applicable to the preparation of other porous catalysts.

## INTRODUCTION

**Scheme 1.**
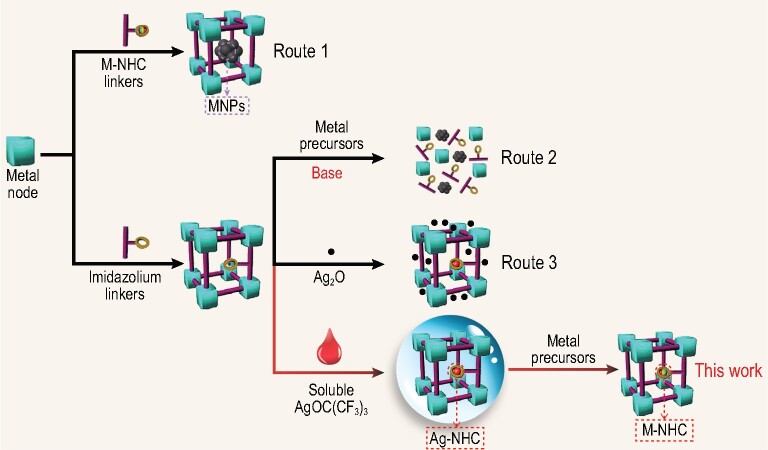
Preparation of M-NHC-functionalized MOFs.

As a class of important organometallic compounds, metal *N-*heterocyclic carbene (M-NHC) complexes have been used widely in medicinal chemistry, material chemistry and chemical catalysis [1–3]. Compared to conventional phosphine ligands, NHC ligands with a σ-electron-donating feature cause M-NHC complexes to be more active and more stable during catalysis. Such complexes have thus been widely employed as highly active homogeneous catalysts for various chemical reactions, including C–C coupling reactions [4], hydrogen transfer reactions [5], olefin metatheses [6] and cycloaddition reactions [7]. However, homogeneous M-NHC catalysts often suffer from deactivation and problems related to catalyst separation and recovery from the reaction systems for reuse. Heterogenization of homogeneous M-NHC catalysts on porous materials has been recognized as a promising alternative means of addressing these problems. Typically, there are two synthetic methods (Scheme 1) for loading M-NHC catalysts into porous materials: encapsulation of M-NHC complexes in the pores of porous materials or M-NHCs covalently bound to the walls of porous materials. Simply loading M-NHC guests into the confined pores of host frameworks, however, can lead to their aggregation and leaching of active components during catalysis. M-NHC complexes covalently grafted onto the skeletons of porous materials could avoid these drawbacks, but early attempts to graft M-NHC complexes covalently onto traditional inorganic or organic porous materials such as silica or amorphous polymers were largely limited by low site densities and low surface areas [8].

During the past two decades, construction of metal-organic frameworks (MOFs) using functional linkers and metal nodes has emerged as a promising versatile platform to immobilize active species for highly efficient catalysis due to their crystalline nature, high surface area, tunable composition and pore structure [9–13]. The functional linkers of MOFs in particular can be employed as grafting sites to covalently anchor M-NHC complexes [14,15], which can then serve as active single sites for highly efficient catalysis. MOF hosts with large surface areas would offer well-defined pores to facilitate the diffusion of reactants and transportation of products [16–19]. To date, two synthetic approaches have been published for the preparation of M-NHC-functionalized MOFs, and to describe the direct self-assembly of M-NHC ligands with metal salts [14] (Scheme 1, Route 1) or the post-synthetic modification (PSM) of imidazolium-functionalized MOFs (Scheme 1) [20]. The first approach, however, works only in a few special cases [21] because most of the moisture- and oxygen-sensitive organometallic M-NHC moieties would fail to survive the hydrothermal or solvothermal conditions. Compared with the direct assembly approach, the post-modification of the imidazolium-functionalized MOFs is promising, but usually requires the use of strong bases such as potassium *tert*-butoxide (KO*^t^*Bu) (Scheme 1, Route 2), which can damage most of the MOF structures. Another choice is the use of solid Ag_2_O that could generate Ag-NHC, followed by a transmetalation step to give final M-NHC complexes (Scheme 1, Route 3) [14]. However, neither Ag_2_O or MOFs are soluble in common solvents, and consequently, development of a facile and general approach to prepare M-NHC-functionalized MOFs for highly efficient heterogeneous catalysis is highly desirable.

**Figure 1.**
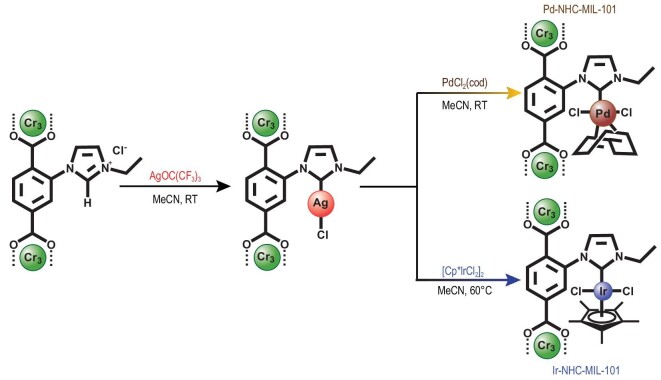
The preparation of Pd-NHC-MIL-101 and Ir-NHC-MIL-101 by the reaction of mesoporous imidazolium-functionalized MIL-101 with the soluble AgOC(CF_3_)_3_ reagent followed by a transmetalation process with PdCl_2_(cod) and [Cp^*^IrCl_2_]_2_, respectively.

Based on the above considerations, discovery of a soluble source of Ag to replace the traditional insoluble Ag_2_O reagent in the preparation of MOFs bonded with M-NHCs could be a feasible strategy. It is known that AgOC(CF_3_)_3_ has good solubility in common aprotic solvents such as CH_2_Cl_2_, and excellent reactivity with imidazolium moieties to form Ag-NHC complexes [22]. This makes it a perfect candidate for the preparation of M-NHC-functionalized MOFs via a transmetalation reaction. As a proof of concept Im-MIL-101, an imidazolium-functionalized mesoporous Cr-MOF was selected as a porous support (Fig. S1) [23,24], with the same topological prototype as MIL-101(Cr) (MIL, Material Institute Lavoisier) [25], to produce the targeted M-NHC-functionalized Im-MIL-101 catalysts, termed M-NHC-MIL-101 (M = Pd, Ir) via a two-step PSM method (Scheme 1). Im-MIL-101 was treated with the soluble silver salt AgOC(CF_3_)_3_ in CH_2_Cl_2_ to generate the Ag-NHC functionalized Im-MIL-101 and this was followed by a transmetalation reaction with a metal salt (Fig. 1). Due to the stable mesoporous frameworks with high surface area, the Pd-NHC-MIL-101 and Ir-NHC-MIL-101 bonded single-site M-NHCs (M = Pd, Ir) that were obtained can be employed as highly active heterogeneous catalysts that promote Suzuki coupling reactions and hydrogen transfer reactions, respectively. The catalysts could be separated from the reaction mixture by simple centrifugation and can be recycled at least three times without obvious loss of activity.

## RESULTS AND DISCUSSION

**Figure 2.**
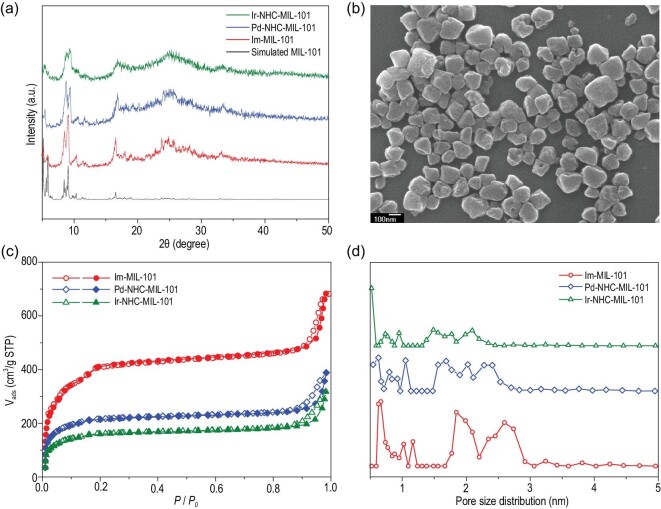
(a) PXRD patterns of the Im-MIL-101, Pd-NHC-MIL-101 and Ir-NHC-MIL-101 that were obtained. (b) The SEM images of Im-MIL-101. Scale bar: 100 nm. (c) N_2_ sorption isotherms at 77 K for Im-MIL-101, Pd-NHC-MIL-101 and Ir-NHC-MIL-101. (d) Pore size distribution of Im-MIL-101, Pd-NHC-MIL-101 and Ir-NHC-MIL-101 based on the density functional theory (DFT) method.

As a chemically stable mesoporous Cr-MOF with large windows, Im-MIL-101 contains abundant imidazolium groups, which make it suitable for the subsequent introduction of single active M-NHC sites. The imidazolium-functionalized mesoporous MOF, Im-MIL-101, was prepared by the solvothermal method described in our previous reports [23,24]. The powder X-ray diffraction (PXRD) patterns of the resulting Im-MIL-101 matched that of the simulated MIL-101(Cr), indicating that the desired Im-MIL-101 had been successfully prepared (Fig. 2a). The composition of Im-MIL-101 was further confirmed by Fourier transform infrared spectroscopy (FT-IR) and X-ray photoelectron spectra (XPS). As shown in Fig. S2, the IR spectra showed the vibrations at 1620 cm^−1^ from the coordinated carboxylic groups of the imidazolium-functionalized linkers in Im-MIL-101 [23]. The XPS in Fig. S3 show peaks centered at 197.3 eV and 198.5 eV attributed to Cl 2P_1/2_ and 2P_3/2_, respectively, and indicating the existence of free Cl^−^ counteranions of the cationic imidazolium motifs in the Im-MIL-101 [26]. The binding energies of N 1s at 401.3 and 399.3 eV were assigned to two chemically different types of nitrogen atoms in the imidazolium of Im-MIL-101 [27]. Notably, the XPS analysis of Cr2p (Fig. S4) revealed the binding energies of the Cr(III) species in Im-MIL-101 to be 577.4 eV and 587.1 eV, indicating a chemical environment similar to that of Cr(III) in MIL-101 [28].

The scanning electron microscopy (SEM) images (Fig. 2b) show that Im-MIL-101 nanoparticles (NPs) were obtained with a diameter between 100 and 300 nm, which could facilitate the contact with substrates and thus promote catalysis efficiency. Furthermore, the N_2_ sorption isotherm analysis revealed that Im-MIL-101 has a high Brunauer Emmer Teller (BET) surface area of 1325 m^2^ g^−1^ and a large pore volume of 1.05 cm^3^/g (Fig. 2c). Notably, the pore size distributions indicated that the open cages with internal free diameters of 18–28 Å still remained (Fig. 2d, Table S2), which would be beneficial for the subsequent reaction with AgOC(CF_3_)_3_ via the PSM process. Thermogravimetric analysis (TGA) revealed that Im-MIL-101 has a high thermal stability and should be stable up to 240°C (Fig. S5). These unique properties make Im-MIL-101 an excellent candidate for the subsequent introduction of single active M-NHC sites and efficient organic catalysis.

The Ag-NHC functionalized Im-MIL-101, termed Ag-NHC-MIL-101, was obtained by the PSM of Im-MIL-101 by treatment with the soluble AgOC(CF_3_)_3_ reagent in MeCN at 25°C for 12 h. Subsequently, the freshly prepared Ag-NHC-MIL-101 was reacted with dichloro(1,5-cyclooctadiene)palladium(II) [PdCl_2_(cod)] in solution in MeCN at 25°C for 48 h affording Pd-NHC-MIL-101. Using the same approach, Ir-NHC-MIL-101 can also be prepared after the reaction of Ag-NHC-MIL-101 and [Cp*IrCl_2_]_2_ at 60^o^C, demonstrating the versatility of this method in the preparation of M-NHC associated porous materials. The PXRD patterns of the obtained Pd-NHC-MIL-101 and Ir-NHC-MIL-101 match well with those of the parent compound Im-MIL-101. These results indicate that their crystalline framework structures were retained after grafting M-NHCs sites via the two-step PSM (Fig. 2a), which highlights the advantages of this mild and facile strategy for the preparation of M-NHC associated porous MOFs. In contrast, it was noticed that the traditional method of using strong bases could destroy the structure of MOFs [29]. Notably, PXRD patterns related to Pd- or Ir-based particles were not observed, suggesting the Pd-NHC and Ir-NHC moieties were not reduced under the PSM conditions [30]. The inductively coupled plasma atomic emission spectroscopy (ICP-AES) revealed that Pd-NHC-MIL-101 and Ir-NHC-MIL-101 have high Pd and Ir contents of 1.47 wt% and 1.31 wt%, respectively. Based on the ICP and elemental analysis results (Table S1), the percentage of M-NHC loadings were ∼10% and ∼7% in Pd-NHC-MIL-101 and Ir-NHC-MIL-101 respectively, with respect to NHC sites. These results indicate that Pd-NHC and Ir-NHC species could be successfully formed and anchored on the pore walls of Im-MIL-101. Although the nitrogen adsorption measurements revealed that Pd-NHC-MIL-101 and Ir-NHC-MIL-101 showed decreased N_2_ adsorption uptake in comparison with Im-MIL-101 (Fig. 2c), they still have large BET surface areas of 657 m^2^/g and 486 m^2^/g, respectively. Despite the cages being partially occupied by Pd-NHC and Ir-NHC moieties, the pore size distribution analysis revealed that Pd-NHC-MIL-101 and Ir-NHC-MIL-101 still have micro- and mesopores of 0.6−2.5 nm (Fig. 2d) and large pore volumes (Table S2), which should permit the diffusion of the reactants and products during catalysis. In addition, the TGA (Fig. S5) displayed that both Pd-NHC-MIL-101 and Ir-NHC-MIL-101 still have high thermal stability up to ∼240^o^C under a N_2_ atmosphere, which is beneficial for their subsequent catalytic applications.

**Figure 3.**
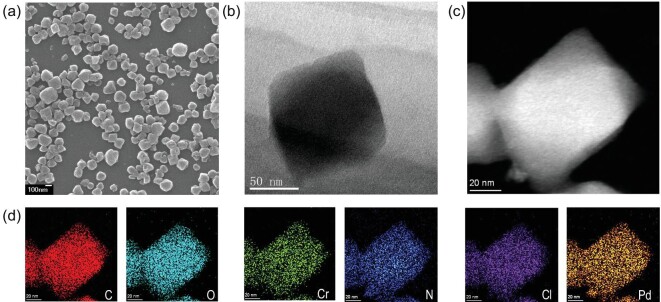
(a) SEM image of Pd-NHC-MIL-101. Scale bar: 100 nm. (b) TEM image of Pd-NHC-MIL-101. (c) HAADF-STEM image and (d) corresponding EDS mapping of C, O, Cr, N, Cl and Pd elements in selected areas of Pd-NHC-MIL-101 as in (c).

The SEM images in Fig. 3a show that Pd-NHC-MIL-101 possesses a nanoscale size similar to that of the parent Im-MIL-101 between 100–300 nm, which could facilitate its contact with the substrate, thus promoting catalytic efficiency [31]. Transmission electron microscopy (TEM) and high-angle annular dark-field scanning transmission electron microscopy (HAADF-STEM) were further used to investigate the state of the metal species in the obtained M-NHC-based catalysts. As shown in Fig. 3b, no obvious Pd NPs were observed in the Pd-NHC-MIL-101 sample, further suggesting the Pd-NHC species were not reduced, which is consistent with the PXRD result (Fig. 2a). Notably, the TEM element mapping images demonstrated that Pd, N and Cl elements were homogeneously distributed over the entire architecture (Fig. 3c and d). These results suggest that single Pd-NHC sites were successfully anchored in Im-MIL-101. Similar TEM and HAADF-STEM results were also observed in Ir-NHC-MIL-101 (Fig. S6), indicating the facility of our strategy for preparing targeted M-NHC-associated MOF catalysts.

**Figure 4.**
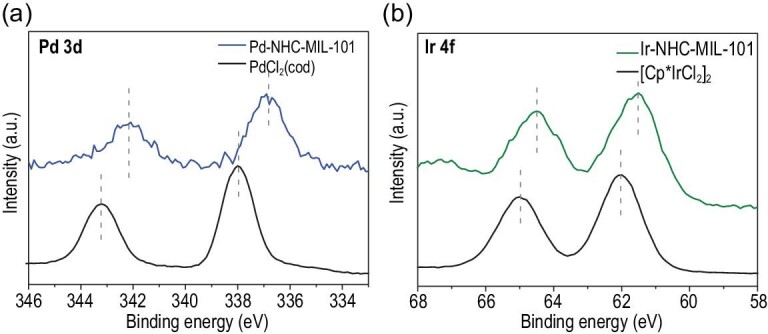
(a) XPS Pd 3d spectra of Pd-NHC-MIL-101 and PdCl_2_(cod). (b) XPS Ir 4f spectra of Ir-NHC-MIL-101 and [Cp^*^IrCl_2_]_2_.

XPS was carried out to confirm the local chemical environment of the Pd single site in Pd-NHC-MIL-101. As shown in Fig. 4a, the binding energies (BEs) of Pd 3d_5/2_ and Pd 3d_3/2_ in Pd-NHC-MIL-101 are 336.9 and 342.1 eV, respectively, indicating that only the Pd(II) species was present with no Pd(0) being produced [32]. Compared with the metal precursor PdCl_2_(cod), significant negative shifts of 1.1 eV from 338.0 eV and 343.2 eV to 336.9 eV and 342.1 eV, respectively, were observed in Pd-NHC-MIL-101. These results indicate that the NHC moieties serve as strong σ donors to coordinate Pd species, which make the Pd species electron-rich, thus promoting the subsequent Suzuki reactions [33]. Similar results were seen in the XPS analysis of Ir-NHC-MIL-101 (Fig. 4b), suggesting the presence of covalently bound, linked Ir-NHC species on the backbones of Ir-NHC-MIL-101.

**Figure 5.**
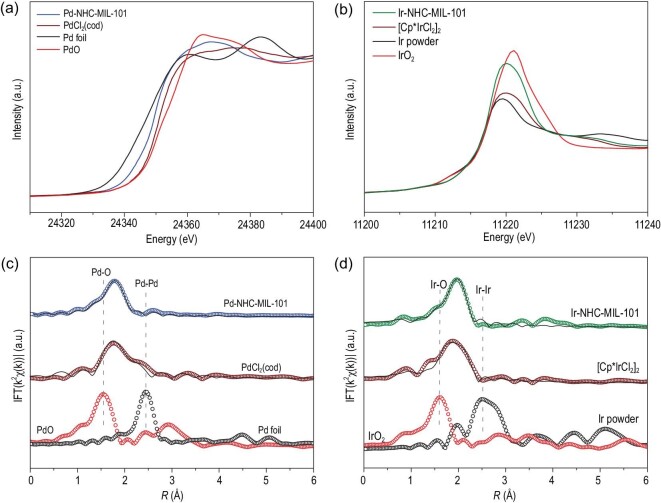
(a) Normalized Pd *K-*edge XANES spectra of the Pd-NHC-MIL-101, PdCl_2_(cod), PdO and Pd foil. (b) Normalized Ir *L*_3-_edge XANES spectra of the Ir-NHC-MIL-101, [Cp^*^IrCl_2_]_2_, IrO_2_ and Ir powder. (c) Fourier transform EXAFS results (open circles) in *R-*space with the corresponding EXAFS fitting results (black solid lines) of the Pd-NHC-MIL-101, PdCl_2_(cod), PdO and Pd foil. (d) Fourier transform EXAFS results (open circles) in *R-*space with the corresponding EXAFS fitting results (black solid lines) of the Ir-NHC-MIL-101, [Cp^*^IrCl_2_]_2_, IrO_2_ and Ir powder.

Synchrotron-based X-ray absorption spectroscopy was performed to confirm the coordination environment of these single-site M-NHC species in Pd-NHC-MIL-101 and Ir-NHC-MIL-101. As shown in the X-ray absorption near-edge structure (XANES) of the Pd *K*-edge in Pd-NHC-MIL-101 (Fig. 5a), the Pd absorption edge position of Pd-NHC-MIL-101 is located in between Pd foil and PdO, indicating that the Pd-NHC single-site species carries positive charge between the metallic Pd(0) and fully oxidized state Pd(II) [34]. When compared with PdCl_2_(cod), the Pd absorption edge of the white line in Pd-NHC-MIL-101 shifted to lower energy, which suggested that some electrons were transferred from NHC to the Pd species, consistent with the XPS analysis [35]. In the Ir *L*_3_-edge XANES profiles in Fig. 5b, the absence of the pre-edge at around 11 212 eV of Ir-NHC-MIL-101 indicates that its coordination environment is different from that of Ir powder or IrO_2_. The white line intensity of Ir-NHC-MIL-101 in Ir *L*_3__-_edge XANES spectrum is situated between those of Ir foil and IrO_2_, indicating that the positively charged iridium is between Ir(0) and Ir (IV) in Ir-NHC-MIL-101 [36].

The coordination environment of the M-NHC species in the catalysts was further analyzed by extended X-ray absorption fine structure (EXAFS). As shown in Fig. 5c in the Pd *R-*space of Pd-NHC-MIL-101, a prominent peak located at ca. 1.8 Å can be ascribed to the Pd-C/Cl scattering path, which is similar to that of PdCl_2_(cod) (1.77 Å) [37]. This result suggested that a Pd species had been grafted to Im-MIL-101 with covalently bound Pd-NHCs. More importantly, no obvious signals assigned to Pd-Pd (2.45 Å) and Pd-O (1.54 Å) were detected, further showing that isolated single-site Pd-NHC species are predominant and no Pd NPs or PdO were formed [38], which is consistent with the TEM and XPS results (Figs 3 and 4). In order to accurately determine the coordination environment of Pd, the fitting results from EXAFS show that the coordination number of Pd species in Pd-NHC-MIL-101 can be calculated to be 5.0 (Fig. S7 and Table S3). This result agrees well with the XANES spectra (Fig. 5a) and is different from the coordination number of the precursor PdCl_2_(cod), proving that the Pd-NHC single sites had been inserted into the pore walls of Pd-NHC-MIL-101 [39]. The same analysis with Ir-NHC-MIL-101 (Fig. 5d) also showed that Ir NPs and IrO_2_ were absent, and revealed that the Ir single site adopted six coordination structures in Ir-NHC-MIL-101 (Fig. S7 and Table S3).

EXAFS wavelet transform (WT) analysis was also performed for further confirmation of the single-site M-NHC species, due to its capability of discriminating between the backscattering atoms by providing radial distance resolution and *k-*space resolution [39]. From the EXAFS WT contour plots of Pd foil (Fig. S8), only one intensity maximum at 9.6 Å^−1^, ascribed to the Pd–Pd bond, was observed. The single intensity maximum at 5.0 Å^−1^ in PdO is standard, and can be ascribed to the Pd–O bond. Notably, the WT analysis of Pd-NHC-MIL-101 reveals only one intensity maximum at ∼6.3 Å^−1^, which is assigned to the contributions from Pd–C. No intensity maximum corresponding to Pd–Pd or Pd–O bonding was observed in Pd-NHC-MIL-101, further demonstrating that single-site Pd-NHC species were generated. The WT analysis for Ir-NHC-MIL-101 is also shown in Fig. S8, and proves intuitively that the Ir species in Ir-NHC-MIL-101 and [Cp^*^IrCl_2_]_2_ are in different states, further suggesting that the generated Ir-NHC single sites were covalent bonded with Ir-NHC-MIL-101 [40].

Inspired by the improved performance of the unique structures of M-NHC-MIL-101 (M = Pd, Ir), two typical model organic reactions were selected to evaluate their catalytic performance. The palladium-catalyzed Suzuki–Miyaura cross-coupling reaction represents a very important carbon–carbon coupling reaction in organic chemistry [41]. The Suzuki–Miyaura coupling reaction of arylboronic acids and aryl halides was thus chosen as a model reaction with which to investigate the catalytic activity and recyclability of Pd-NHC-MIL-101. The catalytic results are summarized in Table 1. Interestingly, in the presence of Pd-NHC-MIL-101 (Pd 1.47 wt%), various aryl halides (Table 1, entries 1−5), including the relatively inert 1-bromo-4-methoxybenzene (Table 1, entry 4), could be smoothly converted to the cross–coupled biaryl products in yields from 91% to 99% within 3 h at 60°C under ambient atmosphere. The less active chlorobenzenes (Table 1, entry 6) also gave a high yield of 90% within 6 h at 80°C. In contrast, when the reaction was carried out in the presence of Im-MIL-101, no desired coupling product was detected under the same reaction conditions (Table 1, entry 7), suggesting that the Pd-NHC moiety was the active site for the Suzuki–Miyaura coupling reactions. Moreover, compared with the high yield of 95% achieved by Pd-NHC-MIL-101 (Table 1, entry 1), a yield of only 41% was obtained for the target product by using a homogeneous PdCl_2_(cod) catalyst under the same reaction conditions (Table 1, entry 8), and a moderate yield of 73% was afforded when a physical mixture of Im-MIL-101 and PdCl_2_(cod) (Table 1, entry 9) was used, indicating the important role of Pd-NHC species inserted on the pore walls of the MOF as a single site promoting the catalytic reaction. Therefore, the high activity of Pd-NHC-MIL-101 can be reasonably attributed to the accessibility of spatially isolated active Pd-NHC single sites through its micro–and mesoporous pores. Such excellent catalytic activity for Pd-NHC-MIL-101 with a turnover frequency (TOF) value of 330 h^−1^ (Table 1, entry 2) caused Pd-NHC-MIL-101 to rank among the most active heterogeneous catalysts for the reported Suzuki–Miyaura coupling reactions of arylboronic acids and aryl halides under atmosphere conditions [41].

**Table 1.** Catalytic Suzuki–Miyaura coupling reactions by Pd-NHC-MIL-101.^a^

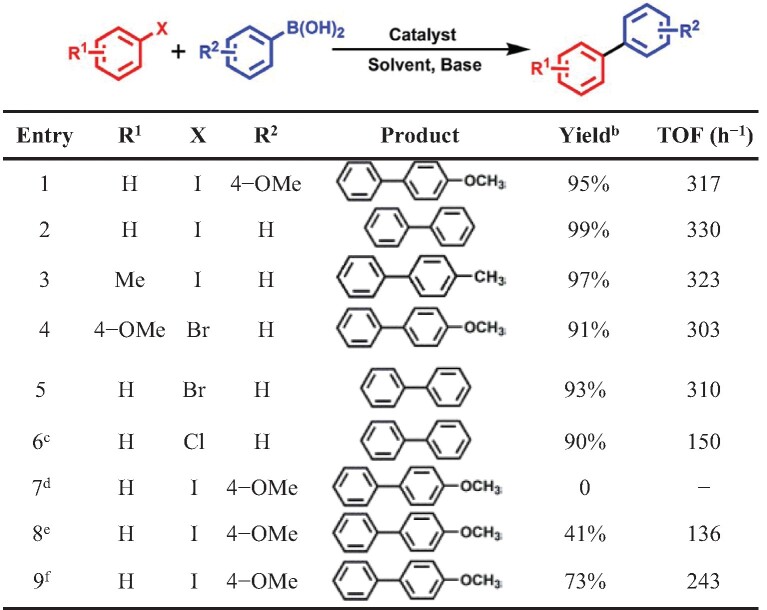

The unique features of the mesoporous MOF with covalently bound M-NHC single sites could enhance the liquid phase catalytic reaction efficiency, and this was also verified by a transfer hydrogenation reaction. This hydrogenation reaction, which is usually catalyzed by iridium complexes, could be improved by avoiding the use of high pressure hydrogen to reduce multiple bonds [42]. Typically, the transfer hydrogenation reactions catalyzed by Ir-NHC-MIL-101 were carried out in isopropanol, which also served as a hydrogen source. A low (5 mol%) concentration of KOH was used as a base and the reaction was performed at 80°C under an ambient atmosphere. In the primary study, acetophenone was selected as a substrate in the model reaction. Interestingly, Ir-NHC-MIL-101 (Ir 1.31 wt%) showed excellent catalytic activity for the conversion of acetophenone to 1-phenylethanol in only 3 h (Table 2, entry 1). Halogen-containing substrates like 4-fluoro- or 4-chloro-substituted acetophenone, also gave high yields of the corresponding alcohols and no dehalogenated side product was detected (Table 2, entries 3 and 4). Sterically demanding substrates such as isobutyrophenone and pivalophenone could also be successfully converted to the corresponding alcohols. In contrast, the reaction failed when Im-MIL-101 was used (Table 2, entry 9), suggesting that Ir-NHC moieties are the active sites for the transfer hydrogenation reaction. In comparison with the high yield of 92% obtained for the 1-phenylethanol, only 35% and 62% yields were obtained over the homogeneous [Cp^*^IrCl_2_]_2_ and a physical mixture of Im-MIL-101 and [Cp^*^IrCl_2_]_2_, respectively (Table 2, entries 7 and 8). These results indicate that the highly distributed Ir-NHC single sites in the mesoporous Ir-NHC-MIL-101 play a key role in efficient catalysis of hydrogenation reactions.

**Table 2.** Catalytic transfer hydrogenation reactions by Ir-NHC-MIL-101.^a^

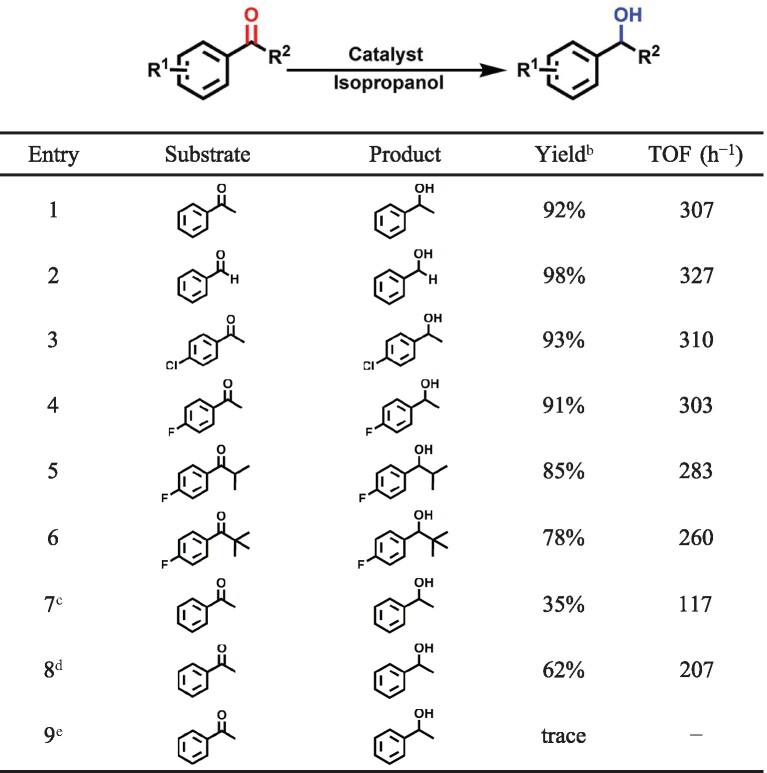

We noticed that, compared with other heterogeneous catalysts [43], the lower required amount of catalyst (0.1 mol% Ir) and the shorter reaction time highlight the advantages of using an M-NHC single-site catalyst to improve catalytic performance [44–46].

Because the Cr-based MOF materials including MIL-101 have good chemical stability in various organic solvents, water, and even under weak basic conditions with protic solvents [25,47], M-NHC-MIL-101 could be recovered and reused. To confirm whether the reactions involved heterogeneous catalysis, a series of control experiments were performed (Fig. S9). After the reaction proceeded for 1 h, the catalyst Pd-NHC-MIL-101 or Ir-NHC-MIL-101 was removed from the hot reaction mixture, and the isolated solution did not exhibit any further reactivity under the same reaction conditions. Moreover, after the reactions were complete, the ICP-AES analysis revealed that almost no Pd or Ir leached out into the isolated solution, which attests to the excellent stability of the covalently bound M-NHC catalyst. The reusability of Pd-NHC-MIL-101 and Ir-NHC-MIL-101 was further examined. As shown in Fig. S10, both of these catalysts could be used at least three times without any obvious loss of catalytic activity and selectivity, which demonstrated the excellent chemical and thermodynamic stability of M-NHC-MIL-101 (M = Pd, Ir). The PXRD patterns showed that the structural integrity and crystalline nature were still maintained after catalysis (Fig. S11). Besides, the N_2_ sorption isotherm analysis and the pore size distribution analysis revealed that these recovered catalysts retain their porous structure (Fig. S12). The SEM (Fig. S13) and HAADF-STEM (Fig. S14) images of the recovered M-NHC-MIL-101 catalysts indicated that the material morphologies were still retained. No obvious metal nanoparticles (MNPs) were observed in their TEM images, suggesting that the corresponding metal species were still highly dispersed in M-NHC-MIL-101. XPS analysis showed that only Pd(II) and Ir(III) species were present in the corresponding recycled catalysts (Fig. S15), which is consistent with the TEM results (Fig. S14).

## CONCLUSION

In summary, we have developed a facile and general approach to the preparation of functionalized MOF catalysts with a covalently anchored and well-defined M-NHC single site by using a soluble AgOC(CF_3_)_3_ source and a subsequent transmetalation reaction. This unique strategy could efficiently avoid the formation of MNPs and destruction of the framework structure of MOFs during the preparation process. The single-site M-NHCs associated with Pd-NHC-MIL-101 and Ir-NHC-MIL-101 that were obtained not only show enhanced catalytic activity compared to the homogeneous metal precursors, but also can be readily used for a broad scope of substrates in Suzuki reactions and transfer hydrogenation reactions. This facile synthesis strategy is widely applicable to the preparation of other porous materials such as covalent organic frameworks, metal organic cages and porous organic polymers with covalently bound M-NHC single sites, for highly efficient catalysis.

## Supplementary Material

nwab157_Supplemental_File

## References

[1] Li Y , Yu JG, Ma LL et al. Strategies for the construction of supramolecular assemblies from poly-NHC ligand precursors. Sci China Chem 2021; 64: 701–18. 10.1007/s11426-020-9937-4

[2] Liu YY , Li WB, Zhang JL. Chiral ligands designed in China. Natl Sci Rev 2017; 4: 326–58. 10.1093/nsr/nwx064

[3] Gou XX , Liu T, Wang YY et al. Ultrastable and highly catalytically active N-heterocyclic-carbene stabilized gold nanoparticles in confined spaces. Angew Chem Int Ed 2020; 59: 16683–9. 10.1002/anie.202006569 32533619

[4] Lu Y , Liu D, Lin YJ et al. Self-assembly of metalla[3]catenanes, Borromean rings and ring-in-ring complexes using a simple π-donor unit. Natl Sci Rev 2020; 7: 1548–56. 10.1093/nsr/nwaa164 34691487 PMC8290965

[5] Wang YM , Lorenzini F, Rebros M et al. Combining bio- and chemo-catalysis for the conversion of bio-renewable alcohols: homogeneous iridium catalysed hydrogen transfer initiated dehydration of 1,3-propanediol to aldehydes. Green Chem 2016; 18: 1751–61. 10.1039/C5GC02157J

[6] Correa A , Cavallo L. The elusive mechanism of olefin metathesis promoted by (NHC) Ru-based catalysts: a trade between steric, electronic, and solvent effects. J Am Chem Soc 2006; 128: 13352–3. 10.1021/ja064924j 17031936

[7] Xia YY , Chen LY, Lv S et al. Microwave-assisted or Cu-NHC-catalyzed cycloaddition of Azido-disubstituted alkynes: bifurcation of reaction pathways. J Org Chem 2014; 79: 9818–25. 10.1021/jo5011262 25268332

[8] Price GA , Hassan A, Chandrasoma N et al. Pd-PEPPSI-IPent-SiO_2_: a supported catalyst for challenging Negishi coupling reactions in flow. Angew Chem Int Ed 2017; 56: 13347–50. 10.1002/anie.201708598 28884491

[9] Li XF , Zhu QL. MOF-based materials for photo- and electrocatalytic CO_2_ reduction. EnergyChem 2020; 2: 100033. 10.1016/j.enchem.2020.100033

[10] Huang YB , Liang J, Wang XS et al. Multifunctional metal-organic framework catalysts: synergistic catalysis and tandem reactions. Chem Soc Rev 2017; 46: 126–57. 10.1039/C6CS00250A 27841411

[11] Huang Q , Liu J, Feng L et al. Multielectron transportation of polyoxometalate-grafted metalloporphyrin coordination frameworks for selective CO_2_-to-CH_4_ photoconversion. Natl Sci Rev 2020; 7: 53–63. 10.1093/nsr/nwz096 34692017 PMC8288839

[12] Hu XJ , Li ZX, Xue H et al. Designing a bifunctional Brønsted acid-base heterogeneous catalyst through precise installation of ligands on metal–organic frameworks. CCS Chem 2019; 1: 616–22.

[13] Zou YH , Huang YB, Si DH et al. Porous metal-organic framework liquids for enhanced CO_2_ adsorption and catalytic conversion. Angew Chem Int Ed 2021; 60: 20915–20 10.1002/anie.202107156. 34278674

[14] Oisaki K , Li QW, Furukawa H et al. A metal-organic framework with covalently bound organometallic complexes. J Am Chem Soc 2010; 132: 9262–4. 10.1021/ja103016y 20557041

[15] Wang H , Shi Z, Yang J et al. Docking of Cu(I) and Ag(I) in metal-organic frameworks for adsorption and separation of xenon. Angew Chem Int Ed 2021; 60: 3417–21. 10.1002/anie.202015262 33247510

[16] Liang F , Wang KY, Lv XL et al. Hierarchically porous metal–organic frameworks: synthetic strategies and applications. Natl Sci Rev 2020; 7: 1743–58. 10.1093/nsr/nwz170 34691505 PMC8290954

[17] Xiong WF , Li HF, You HH et al. Encapsulating metal organic framework into hollow mesoporous carbon sphere as efficient oxygen bifunctional electrocatalyst. Natl Sci Rev 2020; 7: 609–19. 10.1093/nsr/nwz166 34692080 PMC8288918

[18] Cai GR , Ding ML, Wu QY et al. Encapsulating soluble active species into hollow crystalline porous capsules beyond integration of homogeneous and heterogeneous catalysis. Natl Sci Rev 2020; 7: 37–45. 10.1093/nsr/nwz147 34692015 PMC8288971

[19] Wang C , Zhou DD, Gan YW et al. A partially fluorinated ligand for two super-hydrophobic porous coordination polymers with classic structures and increased porosities. Natl Sci Rev 2021; 8: nwaa094. 10.1093/nsr/nwaa094 34691585 PMC8288338

[20] Zhang XJ , Veacheslav V, Feng XW et al. Influence of guest exchange on the magnetization dynamics of dilanthanide single-molecule-magnet nodes within a metal-organic framework. Angew Chem Int Ed 2015; 54: 9861–5. 10.1002/anie.201503636 26119180

[21] Lin IJB , Vasam CS. Preparation and application of N-heterocyclic carbene complexes of Ag(I). Coord Chem Rev 2007; 251: 642–70. 10.1016/j.ccr.2006.09.004

[22] Maishal TK , Basset JM, Boualleg M et al. AgOC(CF_3_)_3_: an alternative and efficient reagent for preparing transition metal-NHC-carbene complexes. Dalton Trans 2009; 35: 6956–9. 10.1039/b900136k 20449136

[23] Liang J , Xie YQ, Wang Q et al. An imidazolium-functionalized mesoporous cationic metal-organic framework for cooperative CO_2_ fixation into cyclic carbonate. Chem Commun 2018; 54: 342–5. 10.1039/C7CC08630J 29182177

[24] Zou YH , Liang J, He C et al. A mesoporous cationic metal-organic framework with high density of positive charge for enhanced removal of dichromate from water. Dalton Trans 2019; 48: 6680–4. 10.1039/C9DT01622H 31050349

[25] Ferey G , Mellot-Draznieks C, Serre C et al. A chromium terephthalate-based solid with unusually large pore volumes and surface area. Science 2005; 309: 2040–2. 10.1126/science.1116275 16179475

[26] Liu TT , Xu R, Yi JD et al. Imidazolium-based cationic covalent triazine frameworks for highly efficient cycloaddition of carbon dioxide. ChemCatChem 2018; 10: 2036–40. 10.1002/cctc.201800023

[27] He C , Wu QJ, Mao MJ et al. Multifunctional gold nanoparticles@imidazolium-based cationic covalent triazine frameworks for efficient tandem reactions. CCS Chem 2020; 2: 2368–80. 10.31635/ccschem.020.202000460

[28] Jiang Z , Xu XH, Ma YH et al. Filling metal-organic framework mesopores with TiO_2_ for CO_2_ photoreduction. Nature 2020; 586: 549–54. 10.1038/s41586-020-2738-2 32906144

[29] Dong Y , Li Y, Wei YL et al. A N-heterocyclic tetracarbene Pd(II) moiety containing a Pd(II)-Pb(II) bimetallic MOF for three-component cyclotrimerization via benzyne. Chem Commun 2016; 52: 10505–8. 10.1039/C6CC04570G 27489026

[30] Chang GG , Ma XC, Zhang YX et al. Construction of hierarchical metal-organic frameworks by competitive coordination strategy for highly efficient CO_2_ conversion. Adv Mater 2019; 31: 1904969−78. 10.1002/adma.201904969 31736178

[31] Qiu X , Chen JM, Zou XW et al. Encapsulation of C–N-decorated metal sub-nanoclusters/single atoms into a metal-organic framework for highly efficient catalysis. Chem Sci 2018; 9: 8962–8. 10.1039/C8SC03549K 30627410 PMC6296299

[32] Yang HQ , Wang YW, Qin Y et al. One-pot preparation of magnetic N-heterocyclic carbene-functionalized silica nanoparticles for the Suzuki–Miyaura coupling of aryl chlorides: improved activity and facile catalyst recovery. Green Chem 2011; 13: 1352–61. 10.1039/c0gc00955e

[33] Pei GX , Liu XY, Wang AQ et al. Ag alloyed Pd single-atom catalysts for efficient selective hydrogenation of acetylene to ethylene in excess ethylene. ACS Catal 2015; 5: 3717–25. 10.1021/acscatal.5b00700

[34] Han LL , Ren ZH, Ou PF et al. Modulating single-atom palladium sites with copper for enhanced ambient ammonia electrosynthesis. Angew Chem Int Ed 2021; 60: 345–50. 10.1002/anie.202010159 32939894

[35] You HH , Wu DS, Chen ZN et al. Highly active and stable water splitting in acidic media using a bifunctional iridium/cucurbit-6-uril catalyst. ACS Energy Lett 2019; 4: 1301–7. 10.1021/acsenergylett.9b00553

[36] Van Velthoven N , Waitschat S, Chavan SM et al. Single-site metal-organic framework catalysts for the oxidative coupling of arenes via C–H/C–H activation. Chem Sci 2019; 10: 3616–22. 30996954 10.1039/c8sc05510f PMC6432273

[37] Pei GX , Liu XY, Yang XF et al. Performance of Cu-alloyed Pd single-atom catalyst for semihydrogenation of acetylene under simulated front-end conditions. ACS Catal 2017; 7: 1491–500. 10.1021/acscatal.6b03293

[38] Wei S , Li A, Liu J-C et al. Direct observation of noble metal nanoparticles transforming to thermally stable single atoms. Nat Nanotechnol 2018; 13: 856–61. 10.1038/s41565-018-0197-9 30013217

[39] Wang DW , Li Q, Han C et al. Atomic and electronic modulation of self-supported nickel-vanadium layered double hydroxide to accelerate water splitting kinetics. Nat Commun 2019; 10: 3899. 10.1038/s41467-019-11765-x 31467288 PMC6715676

[40] Xiao ML , Zhu JB, Li GR et al. A single-atom iridium heterogeneous catalyst in oxygen reduction reaction. Angew Chem Int Ed 2019; 58: 9640–5. 10.1002/anie.201905241 31120620

[41] Biffis A , Centomo P, Del Zotto A et al. Pd metal catalysts for cross-couplings and related reactions in the 21st century: a critical review. Chem Rev 2018; 118: 2249–95. 10.1021/acs.chemrev.7b00443 29460627

[42] Hey DA , Reich RM, Baratta W et al. Current advances on ruthenium(II) N-heterocyclic carbenes in hydrogenation reactions. Coord Chem Rev 2018; 374: 114–32. 10.1016/j.ccr.2018.06.005

[43] Yang YJ , Deng D, Zhang SL et al. Porous organic frameworks featured by distinct confining fields for the selective hydrogenation of biomass-derived ketones. Adv Mater 2020; 32: 1908243. 10.1002/adma.201908243 32323418

[44] Lee JS , Kapustin EA, Pei XK et al. Architectural stabilization of a gold(III) catalyst in metal-organic frameworks. Chem 2020; 6: 142–52. 10.1016/j.chempr.2019.10.022 32285019 PMC7153757

[45] Han YF , Jin GX, Hahn FE. Postsynthetic modification of dicarbene-derived metallacycles via photochemical 2+2 cycloaddition. J Am Chem Soc 2013; 135: 9263–6. 10.1021/ja4032067 23731462

[46] Chen XY , Zhao K, Liu Q et al. N-heterocyclic carbene-catalyzed 1,6-addition of homoenolate equivalent intermediates: asymmetric synthesis of nonspirocyclic quaternary oxindoles. CCS Chem 2019; 1: 261–7. 10.31635/ccschem.019.20190023

[47] Huang YB , Lin ZZ, Cao R. Palladium nanoparticles encapsulated in a metal–organic framework as efficient heterogeneous catalysts for direct C2 arylation of indoles. Chem Eur J 2011; 17: 12706–12. 10.1002/chem.201101705 21956646

