# Supplementary material for: Metal-organic frameworks bonded with metal N-heterocyclic carbenes for efficient catalysis
Source: Natl Sci Rev. 2021 Aug 24;9(6):nwab157. doi: 10.1093/nsr/nwab157 (PMC9270066; doi:10.1093/nsr/nwab157)
Supplement: nwab157_Supplemental_File [file nwab157_supplemental_file.pdf]

Support Information

Metal-Organic Frameworks Bonded with Metal N−Heterocyclic Carbenes for Efficient Catalysis

Chang He[1,2], Jun Liang[1], Yu−Huang Zou[1], Jun−Dong Yi[1], Yuan−Biao Huang[1,2,]*, and Rong Cao[1,2,3,]*

[1] State Key Laboratory of Structural Chemistry, Fujian Institute of Research on the Structure of Matter, Chinese Academy of Sciences, Fuzhou 350002, China

[2] University of Chinese Academy of Science, Beijing 100049, China

[3] Science & Technology Innovation Laboratory for Optoelectronic Information of China, Fuzhou, Fujian, 350108, China

*Corresponding authors. E-mails: ybhuang@fjirsm.ac.cn; rcao@fjirsm.ac.cn

Table of contents:

1. Materials and instrumentation

2. Syntheses

2.1. Synthesis of (Cl^{-})Etim−H_2BDC

2.2. Synthesis of Im−MIL−101

2.3. Synthesis of Pd−NHC−MIL−101 and Ir−NHC−MIL−101

3 Catalyses

3.1. General procedure for Pd−NHC−MIL−101 catalyzed Suzuki−Miyaura reaction.

3.2. General procedure for Ir−NHC−MIL−101 catalyzed transfer hydrogenation.

4. Characterizations

1. Materials and instrumentation

All chemicals were obtained from commercial sources and used without further purification. Powder X−ray diffraction patterns (PXRD) were recorded on a Rigaku Dmax 2500 diffractometer equipped with Cu−Kα radiation (λ = 1.54056 Å) over the 2θ range of 4−50° with a scan speed of 3° min^{-1} at room temperature. N_2 sorption isotherms at 77 K for materials were collected by using a Micrometrics ASAP 2460 instrument. Before the measurement, the samples were evacuated and activated at 423 K under vacuum for 12 hours. The particle size and morphologies of materials were investigated by using transmission electron microscopy (TEM) and high−angle annular dark−field scanning transmission electron microscopy (HAADF−STEM) on Talos−F200X equipped with EDS detector at an accelerating voltage of 200 kV. UV−visible diffuse reflectance spectroscopy was measured on a Shimadzu UV−2600 with $BaSO_4$ as the reference and transformed to the absorption spectra according to the Kubelka−Munk relationship. Infrared (IR) spectra were recorded using KBr pellets on a PerkinElmer Spectrum One in the range of 400−4000 cm^{-1}. Valence state of element was evaluated by X−ray photoelectron spectroscopy (XPS) on an ESCALAB 250Xi X−ray photoelectron spectrometer (Thermo Fisher) using an Al Ka source (15 kV, 10 mA) with Ar etching for 30 min (the charge of C−C carbon species here was corrected to 284.6 eV). The amount of Au in catalyst were quantified by inductively coupled plasma atomic emission spectroscopy (ICP−AES) on an Ultima 2 analyzer (Jobin Yvon). The ^{1}H NMR was performed on AVANCE III Bruker Biospin spectrometer, operating at 400 MHz. The gas chromatography–mass (GC−MS) measurements were performed on a Shimadzu QP−2020 GC−MS system. The gas chromatography (GC) measurements were performed on a G7890A−GC. XAFS spectra at the Pd *K*−edge and Ir L_3−edge were measured at the beamline BL14W1 station of the Shanghai Synchrotron Radiation Facility, China.

2. Syntheses

2.1 Synthesis of (Cl^-)Etim−H_2BDC: (Cl^-)Etim−H_2BDC was synthesized according to our previous literature,[1,2] and then used as the linker for Im−MIL−101.[3]

2.2 Synthesis of Im−MIL−101: [(Cl^-)Etim−H_2BDC] (34 mg, 0.1 mmol) and $CrCl_3$ $6H_2O$ (27 mg, 0.1 mmol) in a 0.5 mL mixed solvent (H_2O/EtOH =1:1) were treated with ultrasonication and then put in a 10 mL Teflon−lined autoclave before keeping the temperature at 180 ℃ for 12 h in an oven. After cooling down to room temperature, green precipitate was harvested by centrifugation. To activate Im−MIL−101, the obtained sample was dispersed in water and sealed in a Teflon at 80 ℃ for 6 h. This was repeated again. Then Im−MIL−101 was dispersed in ethanol and activated at 80 ℃ for 10 h. After removal of ethanol by filtration, the sample was dried at 150 ℃under vacuum for 12 h.

2.3 Synthesis of Pd−NHC−MIL−101 and Ir−NHC−MIL−101

Synthesis of Pd−NHC−MIL−101: In a typical synthesis, 50.0 mg dried Im−MIL−101 was dispersed in 2ml ultra−dry acetonitrile. Then, an ultra−dry CH_2Cl_2 solution (2 mL) containing 9.0 mg (0.025 mmol) $AgOC(CF_3)_3$ was added and stirred for 12 h to produce intermediate Ag−NHC−MIL−101. Subsequently, 2 mL ultra−dry acetonitrile containing 7.1 mg (0.025 mmol) $PdCl_2$(cod) was added into the above solution and continued to be stirred for 48 hours at 25 ℃. The resultant light green solid as Pd−NHC−MIL−101 was filtered and washed with acetonitrile (30 mL), dichloromethane (30 mL), and methanol (30 mL) successively until the filtrate became colorless. Finally, the obtained Pd−NHC−MIL−101 was dried at 120 ℃ under vacuum for further use.

2.4 Synthesis of Ir−NHC−MIL−101: In a typical synthesis, 50 mg dried Im−MIL−101 was dispersed in 2ml of ultra−dry acetonitrile. Then, an ultra−dry CH_2Cl_2 solution (2 mL) containing 9.0 mg (0.025 mmol) $AgOC(CF_3)_3$ was added and stirred for 12 h to produce intermediate Ag−NHC-MIL-101. Subsequently, 2 mL

ultra−dry acetonitrile containing 10 mg (0.013 mmol) $[Cp^*IrCl_2]_2$ was added into the above solution and continued to be stirred at 60 ℃ for 24 hours. The resultant light green solid as Ir−NHC−MIL−101 was filtered and washed with acetonitrile (30 mL), dichloromethane (30 mL), and methanol (30 mL) successively until the filtrate became colorless. Finally, the obtained Ir−NHC−MIL−101 was dried at 120 ℃ under in vacuum for further use.

3. Catalyses

3.1 General procedure for Pd−NHC−MIL−101 catalyzed Suzuki−Miyaura reaction.

Typically, aryl halide (1.0 equiv), arylboronic acid (1.5 equiv), K_2CO_3 (2.0 equiv) and Pd−NHC−MIL−101 (0.1 mol% based on Pd) were stirred in 1,4−dioxane−H_2O (4 mL, v:v = 3:1) in a capped glass bottle at 60 ℃ for 3 h under ambient atmosphere. After the reaction, the mixture was cooled to room temperature, and the catalyst was separated by centrifugation and the solvent was then removed using a rotary evaporator. Ethyl acetate was added to the water layer, washed with brine, dried over $MgSO_4$, filtered and concentrated under vacuo. The product was analyzed by GC−MS.

For the recycle experiment, the catalyst was recovered at the end of catalytic reaction of phenylboronic acid (0.5 mmol)and iodobenzene, and was washed by water (30 mL) and ethyl acetate (30 mL) respectively, followed by drying at 60 ℃ under vacuum. The dried catalyst was then used for the next run.

3.2 General procedure for Ir−NHC−MIL−101 catalyzed transfer hydrogenation.

Typically, aromatic ketone (1.0 equiv), KOH(0.05 equiv) and Ir−NHC−MIL−101 (0.1 mol% based on Ir) were stirred in isopropanol (2.5 mL) in a capped glass bottle at 80 ℃ for 3 h under ambient atmosphere. After the reaction, the mixture was cooled to room temperature, and the catalyst was separated by centrifugation and the solvent was then removed using a rotary evaporator. The product was analyzed by GC−MS.

For the recycle experiment, the catalyst was recovered at the end of catalytic reaction of acetophenone (0.5 mmol), and was washed with acetone (30 mL) and ethyl acetate (30 mL) respectively, followed by drying at 60 ℃ under vacuum. The dried catalyst was then used for the next run.

4. Characterizations

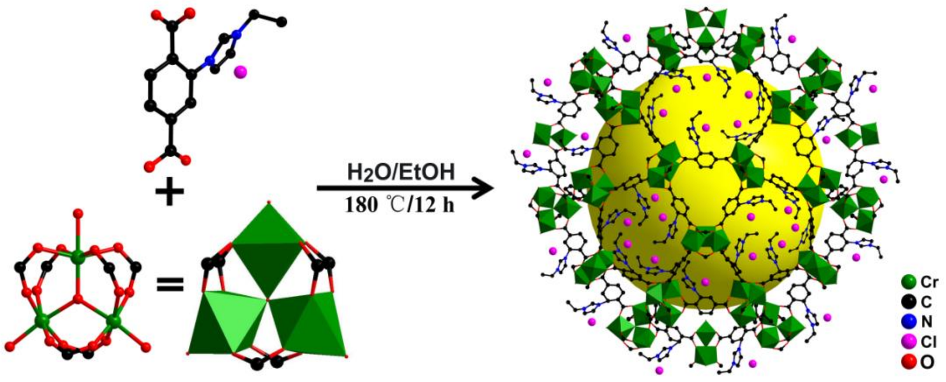

Figure S1. Schematic views for the synthesis of mesoporous imidazolium functionalized Im−MIL−101.[3]

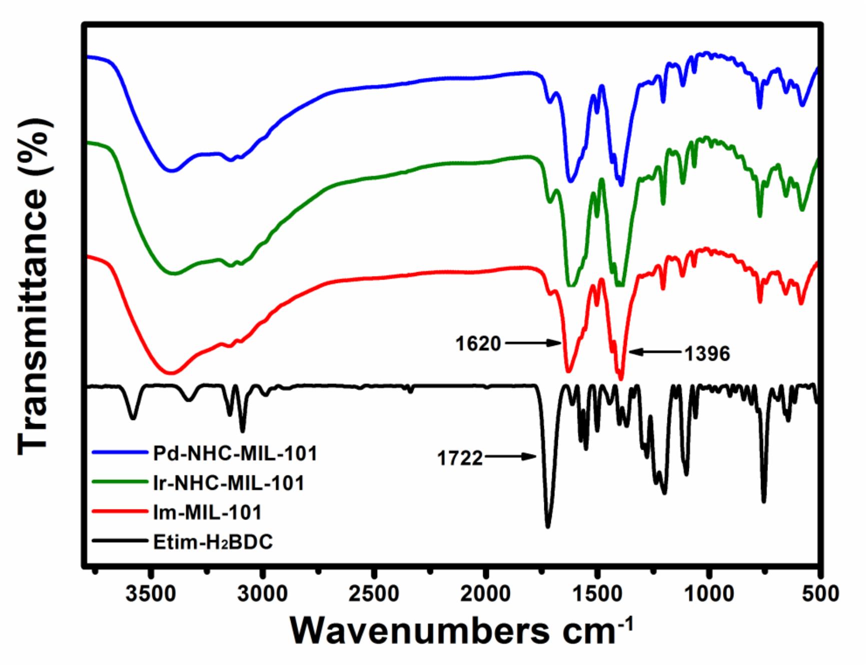

Figure S2. The FT−IR spectra of (Br^{-})Etim−H_2BDC as the ligand of Im−MIL−101,

Ir−NHC−MIL−101 and Pd−NHC−MIL−101.

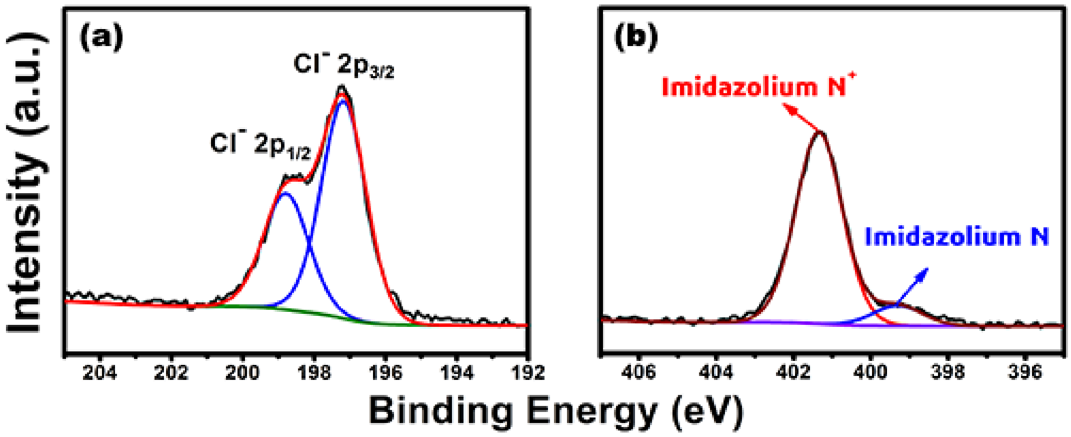

Figure S3. (a) Cl 2p spectra of Im−MIL−101 (b) N 1s spectra of Im−MIL−101.

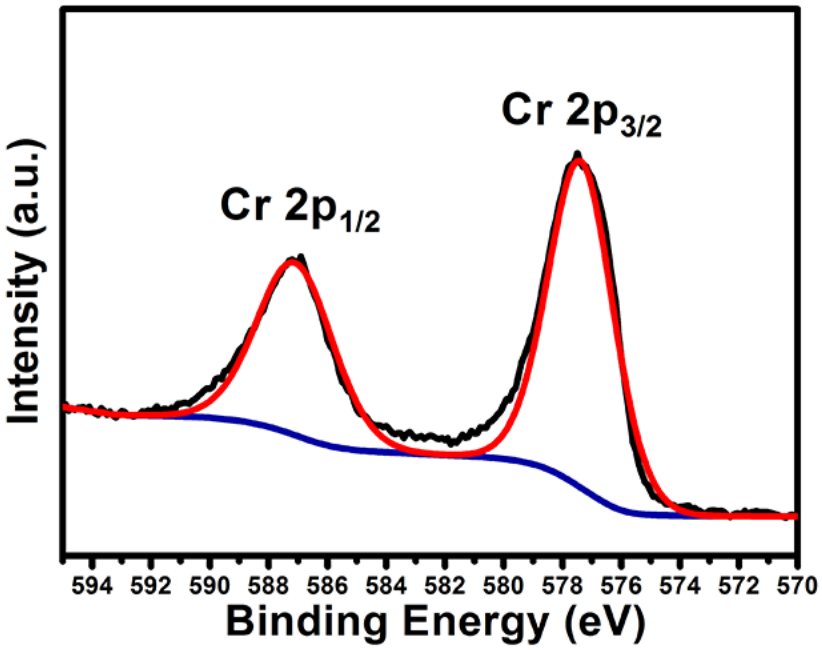

Figure S4. Deconvoluted XPS Cr 2p spectra of Im−MIL−101.

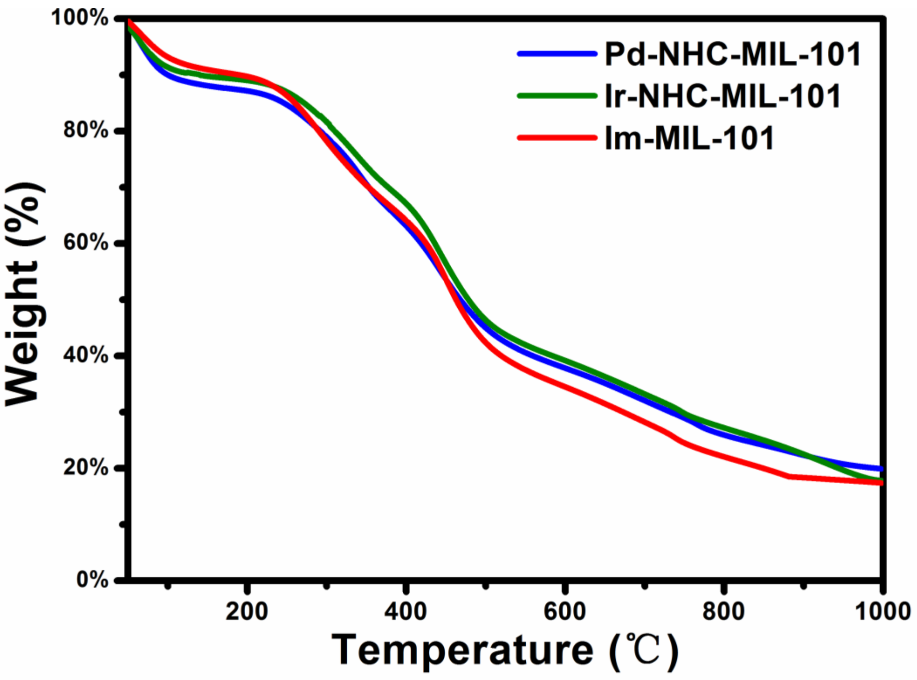

Figure S5. Thermogravimetric analysis of Im−MIL−101, Pd−NHC−MIL−101 and Ir−NHC−MIL−101 under N_2 atmosphere.

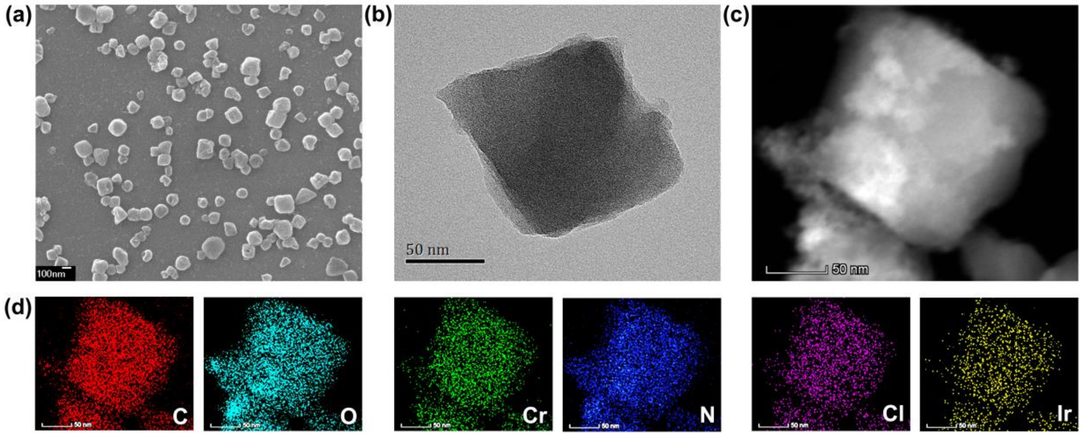

Figure S6. (a) SEM images of Ir−NHC−MIL−101. Scale bar: 100 nm. (b) TEM images of Ir−NHC−MIL−101. (c) HAADF−STEM image and (d) corresponding EDS mapping of C, O, Cr, N, Cl and Ir elements in selected area of Ir−NHC−MIL−101 as in (c).

Table S1. ICP and element analysis of Im−MIL−101, Pd−NHC−MIL−101 and Ir−NHC−MIL−101.

	**Cr**	**C**	**N**	**O**	**Pd**	**Ir**	**Ag**
**Im−MIL−101**	12.8%	40.6%	6.5%	37.8%	−	−	−
**Pd−NHC−MIL−101**	11.5%	39.3%	6.2%	35.4%	1.5%	−	−
**Ir−NHC−MIL−101**	11.8%	38.2%	6.1%	34.1%	−	1.3%	−
**Pd−NHC−MIL−101 (after catalysis)**	11.4%	39.7%	5.2%	32.6%	1.4%	−	−
**Ir−NHC−MIL−101 (after catalysis)**	11.7%	37.9%	5.5%	33.4%	−	1.3%	−

Table S2. Summary of BET surface areas, pore volume and pore size of Im−MIL−101, Pd−NHC−MIL−101 and Ir−NHC−MIL−101.

Sample	BET surface area (m^2/g)	Pore volume (cm^3/g)	Pore size (nm)
**Im−MIL−101**	1325	1.05	0.6−1.2, 1.8−2.9
**Pd−NHC−MIL−101**	657	0.59	0.6−1.1, 1.6−2.5
**Ir−NHC−MIL−101**	486	0.48	0.6−1.0, 1.6−2.3
**Pd−NHC−MIL−101 (after catalysis)**	435	0.45	0.5−1.0, 1.6−2.4
**Ir−NHC−MIL−101 (after catalysis)**	261	0.32	0.5−0.8, 1.5−2.2

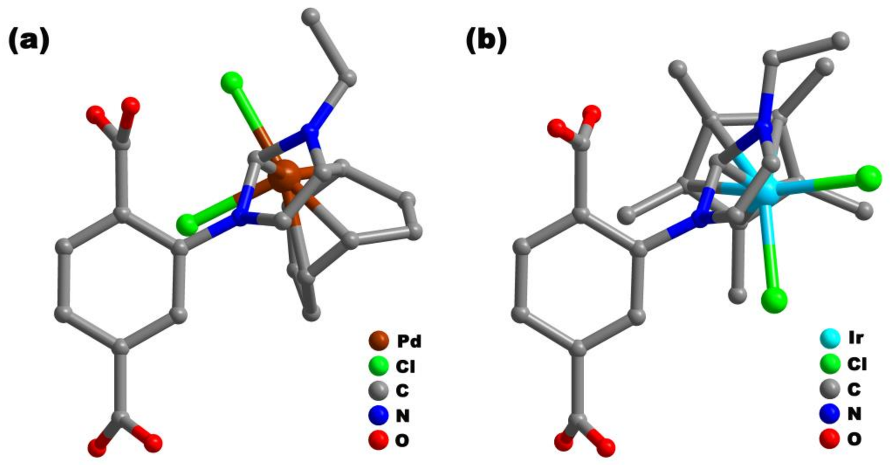

Figure S7. The hypothetical structure model of Pd−NHC and Ir−NHC species in Pd−NHC−MIL−101 and Ir−NHC−MIL−101 respectively. These structures were simulated on Materials Studio software.

Table S3. EXAFS data fitting results of samples.

Sample	Path	CN	R(Å)	$\sigma^2(10^{-3}Å^2)$	R factor
Pd−NHC−MIL−101	Pd−C1(carbene)	1	2.029	3.7±2.9	0.01
	Pd−C2(cod)	2	2.206	4.2±1.9	0.01
	Pd−Cl	2	2.303	2.6±2.5	0.01
$PdCl_2$(cod)	Pd−C(cod)	2	2.207	2.3±3.7	0.02
	Pd−Cl	2	2.312	3.6±4.4	0.02
Ir−NHC−MIL−101	Ir−C1(carbene)	1	2.003	3.2±1.1	0.02
	Ir−C2(Cp*)	3	2.132	5.1±3.6	0.02
	Ir−Cl	2	2.395	4.3±1.7	0.02
$[Cp^*IrCl_2]_2$	Ir−C(Cp*)	3	2.143	6.3±3.2	0.03
	Ir−Cl	3	2.441	5.2±3.8	0.03

CN, coordination number; R, distance between absorber and backscatter atoms; σ^2, Debye−Waller factor (a measure of thermal and static disorder in absorber−scatterer distances); R factor is used to value the goodness of the fitting.

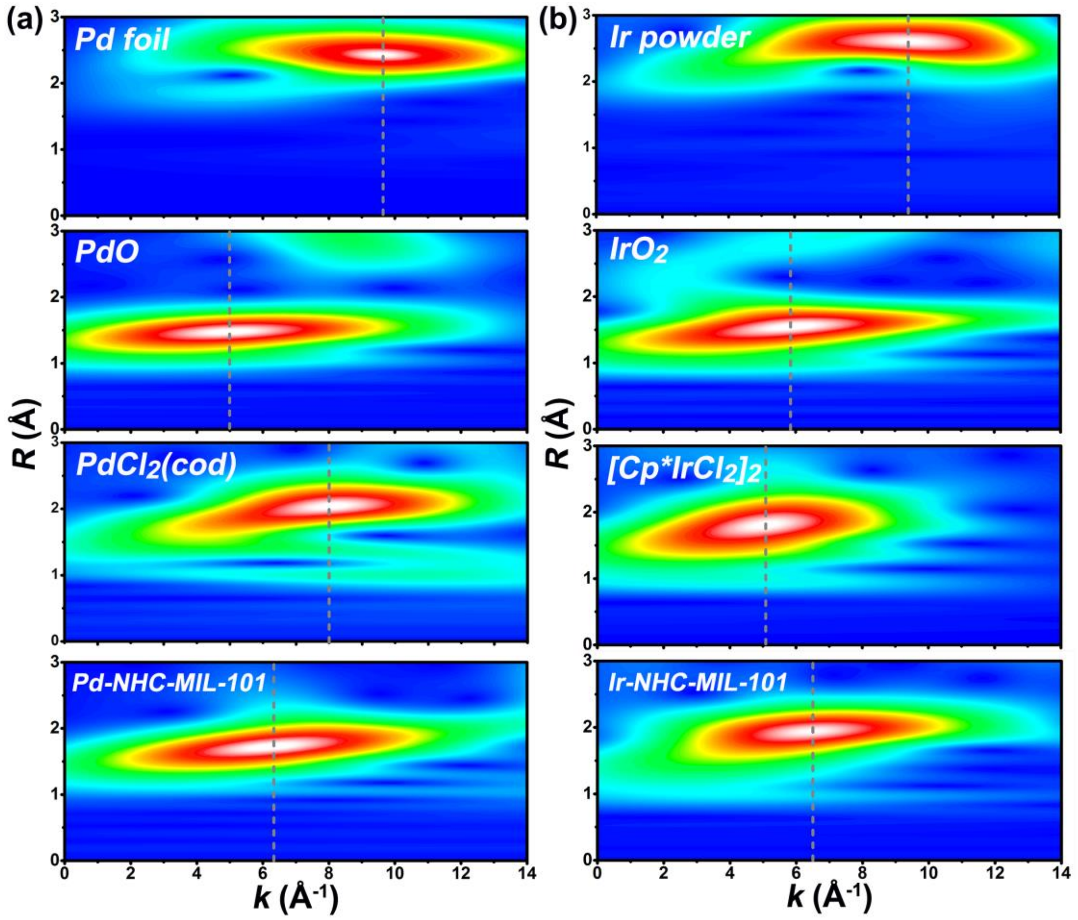

Figure S8. Wavelet transform for the k^2−weighted EXAFS signals of different samples.

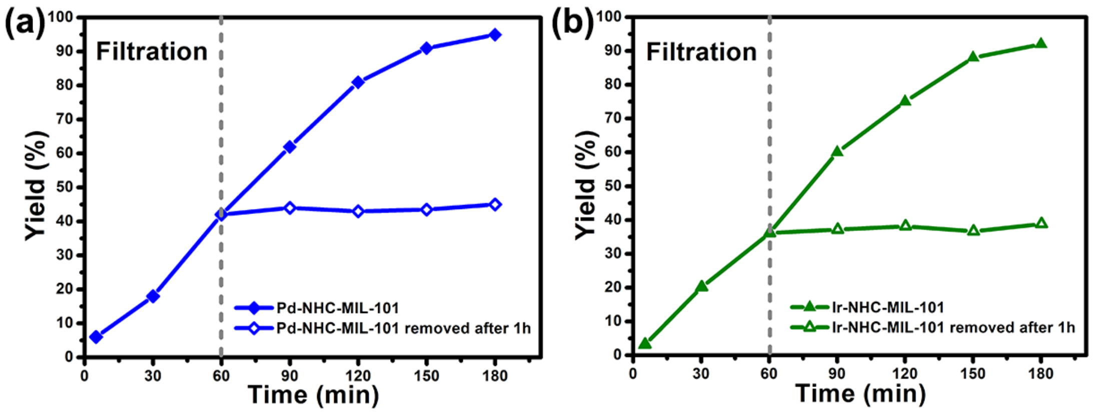

Figure S9. Hot filtration test of (a) Pd−NHC−MIL−101 in the Suzuki−Miyaura coupling reaction of iodobenzene and *p*−methoxybenzeneboronic acid. (b) Ir−NHC−MIL−101 in the transfer hydrogenation reaction of acetophenone.

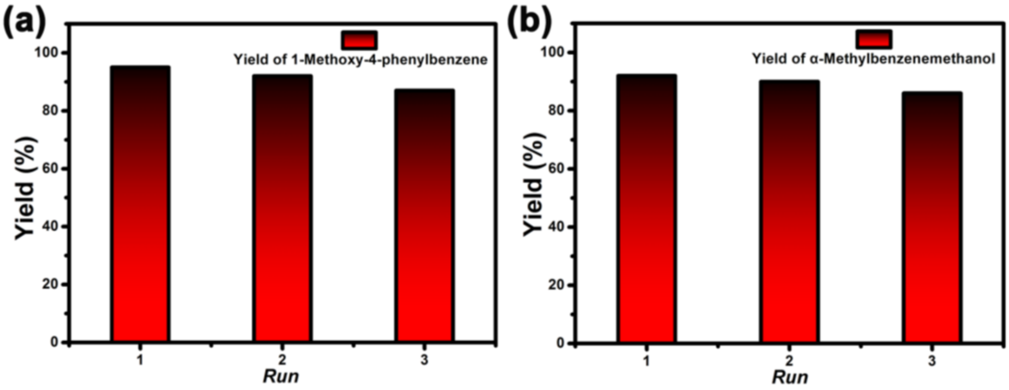

Figure S10. (a) Recycle experiment result over three runs of Pd−NHC−MIL−101 for Suzuki−Miyaura coupling reaction with iodobenzene and *p*−methoxybenzeneboronic acid as reactants. (b) Recycle experiment result over three runs of Ir−NHC−MIL−101 for the transfer hydrogenation reaction of acetophenone.

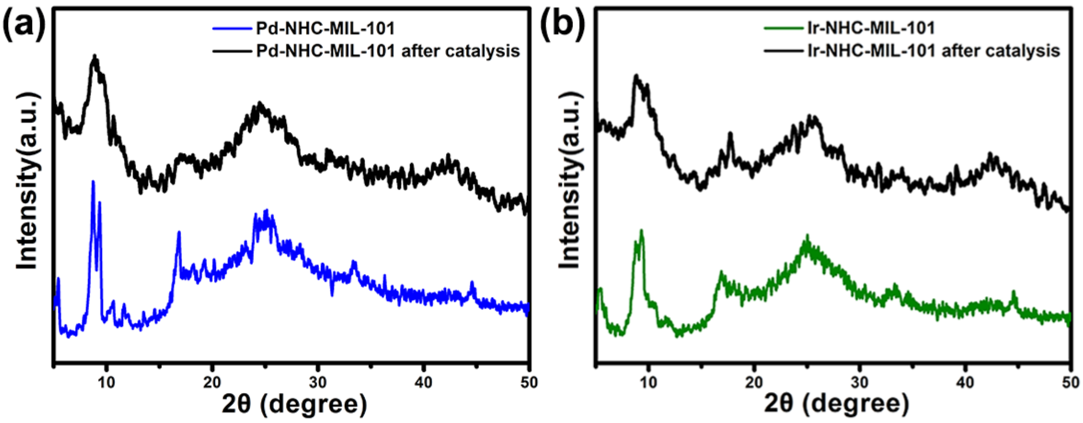

Figure S11. (a) PXRD patterns of Pd−NHC−MIL−101 before and after catalysis. (b) PXRD patterns of Ir−NHC−MIL−101 before and after catalysis.

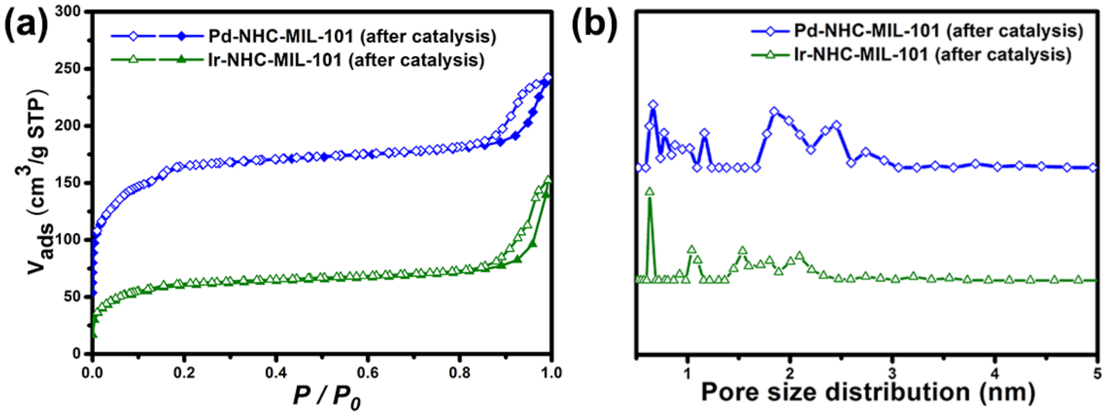

Figure S12. (a) Pore size distributions and (b) N_2 sorption isotherms at 77 K of Pd−NHC−MIL−101 and Ir−NHC−MIL−101 after catalysis, respectively.

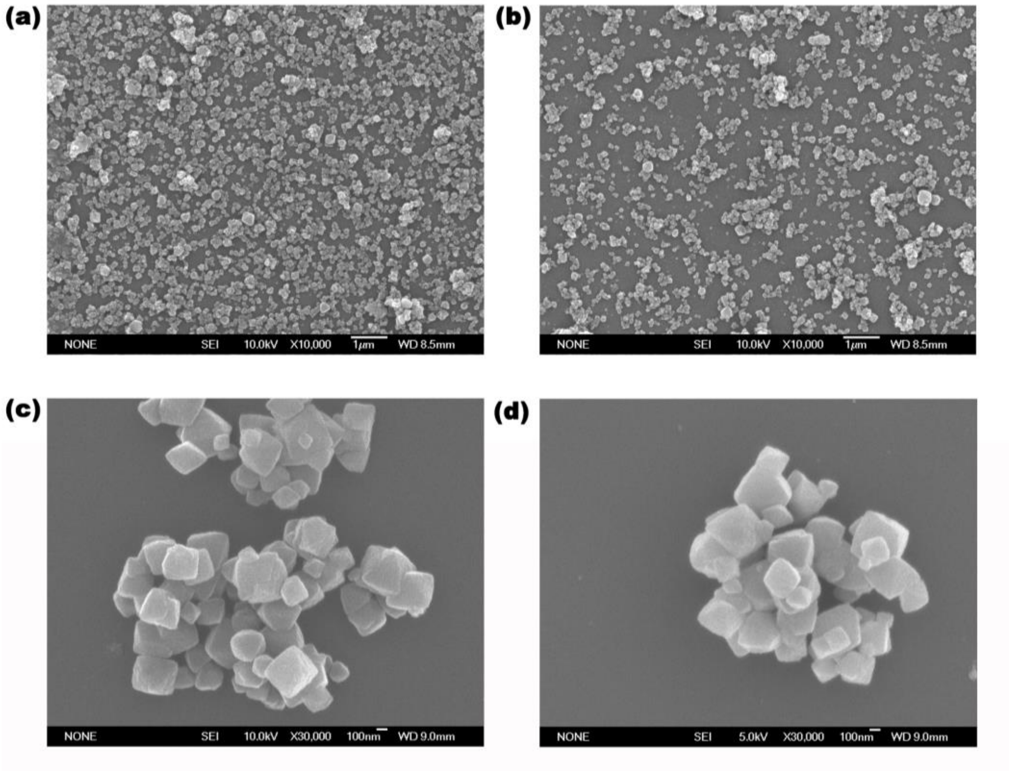

Figure S13. The SEM images of (a) recovered Pd−NHC−MIL−101 and (b) recovered Ir−NHC−MIL−101. The enlarged SEM images of the (c) recovered Pd−NHC−MIL−101 and (d) recovered Ir−NHC−MIL−101

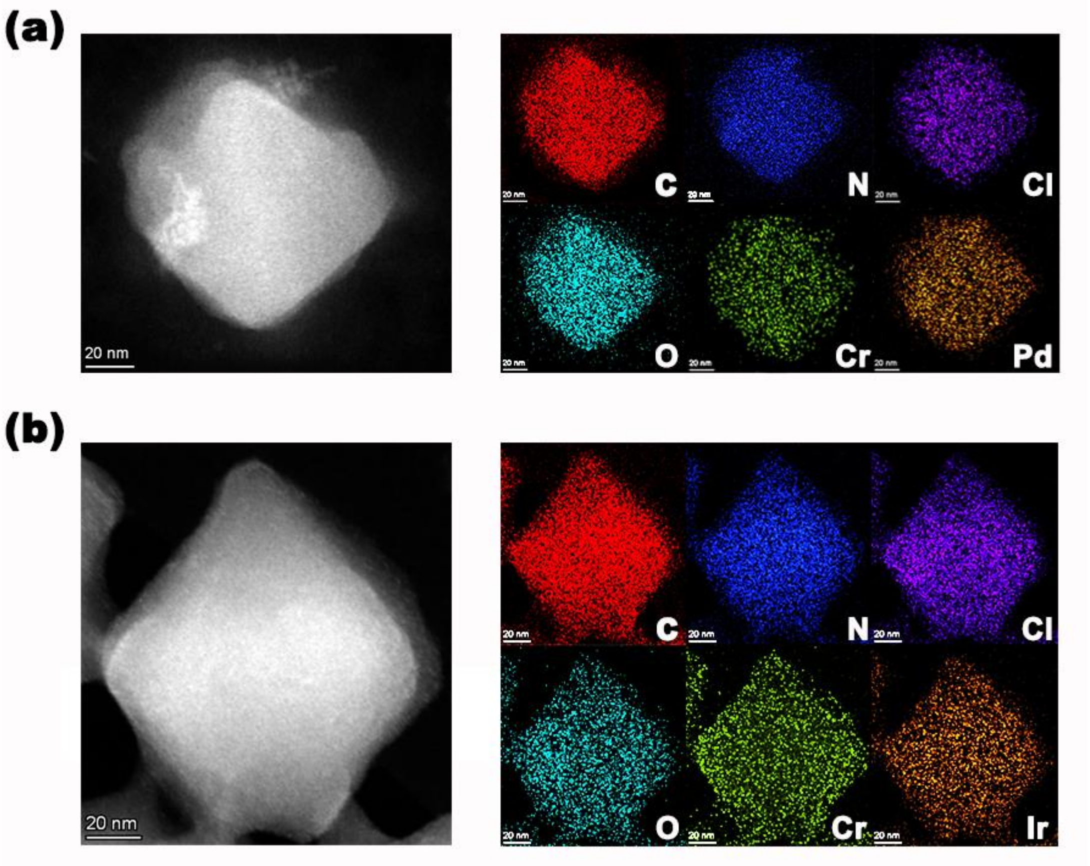

Figure S14. (a) HAADF−STEM image of the recovered Pd−NHC−MIL−101 and corresponding EDS mapping of C, N, Cl, O, Cr and Pd elements in selected area of Pd−NHC−MIL−101. (b) HAADF−STEM image of the recovered Ir−NHC−MIL−101 and corresponding EDS mapping of C, N, Cl, O, Cr and Ir elements in selected area of Ir−NHC−MIL−101.

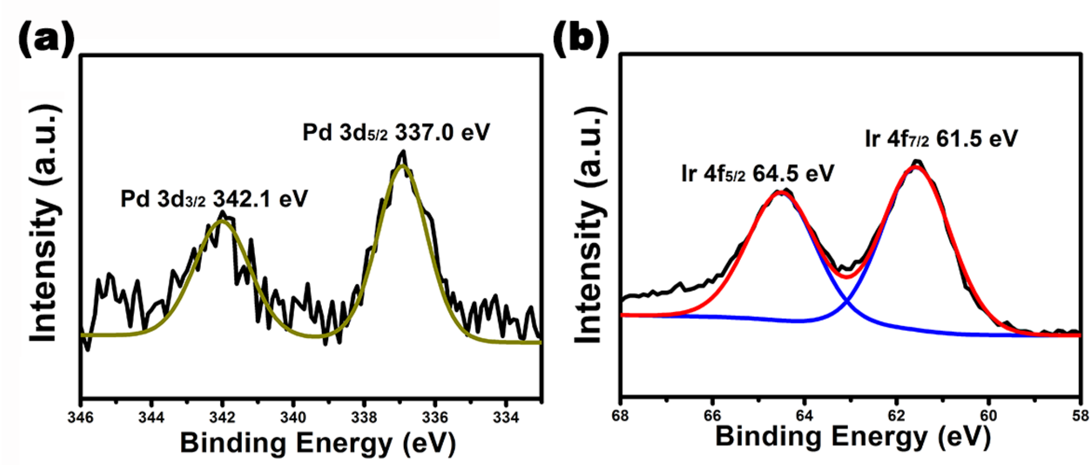

Figure S15. (a) XPS Pd 3d spectra of the recovered Pd−NHC−MIL−101 and (b) XPS

Ir 4f spectra of the recovered Ir−NHC−MIL−101.

Supplementary References

1. Liang J, Chen RP and Wang XY *et al.* Postsynthetic ionization of an imidazole−containing metal−organic framework for the cycloaddition of carbon dioxide and epoxides. *Chem Sci* 2017; **8**: 1570−5.

2. Liang J, Xie YQ and Wang Q *et al*. An imidazolium−functionalized mesoporous cationic metal−organic framework for cooperative CO_2 fixation into cyclic carbonate. *Chem Commun* 2018; **54**: 342−5.

3. Zou YH, Liang J and He C *et al*. A mesoporous cationic metal−organic framework with high density of positive charge for enhanced removal of dichromate from water. *Dalton Trans* 2019; **48**: 6680−4.
